# Multicenter evaluation of planning quality in intracranial stereotactic radiotherapy for brain metastases

**DOI:** 10.1016/j.phro.2026.100919

**Published:** 2026-02-06

**Authors:** Sara Abdollahi, Rachid Boucenna, Cécile Chatelain, Nathan Corradini, Marie Fargier-Voiron, Vincent Fave, Juan Garcia, Sarah Ghandour, Matthias Guckenberger, Käthy Haller, Martin Härtig, Tanja Hertel, Maud Jaccard, Stephan Klöck, Jérôme Krayenbühl, Natacha Ruiz López, Philippe Logaritsch, Peter Pemler, Harald Petermann, Olivier Pisaturo, Francesco Pupillo, Daniel Schmidhalter, Christian Tata, Sheeba Thengumpallil, Veronique Vallet, Patrick Weber, Nicolaus Andratschke, Stephanie Tanadini-Lang

**Affiliations:** a Department of Radiation Oncology, Universitätsspital Zürich, Zürich, Switzerland; b Department of Radiation Oncology, Hirslanden Clinique Bois-Cerf, Lausanne, Switzerland; c Department of Radiation Oncology, Radio Onkologiezentrum Biel Seeland, Biel-Bienne, Switzerland; d Department of Radiation Oncology, Clinica Luganese Moncucco, Lugano, Switzerland; e Department of Radiation Oncology, Clinique de Genolier, Genolier, Switzerland; f Department of Radiation Oncology, Hôpital de La Tour, Meyrin, Switzerland; g Department of Radiation Oncology, Atrys Schweiz AG, Liestal, Switzerland; h Department of Radiation Oncology, Hôpital Riviera-Chablais, Vaud-Valais, Rennaz, Switzerland; i Department of Radiation Oncology, Klinik Hirslonden, Zürich, Switzerland; j Department of Radiation Oncology, St. Claraspital AG, Basel, Switzerland; k Department of Radiation Oncology, Kantonsspital St.Gallen, St.Gallen, Switzerland; l Department of Radiation Oncology, Clinique Générale-Beaulieu, Genève, Switzerland; m Department of Radiation Oncology, Lindenhofspital, Bern, Switzerland; n Department of Radiation Oncology, Hôpitaux Universitaires de Genève, Genève, Switzerland; o Department of Radiation Oncology, Luzerner Kantonsspital, Luzern, Switzerland; p Department of Radiation Oncology, Stadtspital Zürich Triemli, Zürich, Switzerland; q Department of Radiation Oncology, Universitätsspital Basel, Basel, Switzerland; r Department of Radiation Oncology, Hôpital Fribourgeois, Fribourg, Switzerland; s Medical Physics Unit, Imaging Institute of Southern Switzerland, Ente Ospedaliero Cantonale, Bellinzona, Switzerland; t Division of Medical Radiation Physics and Department of Radiation Oncology, Inselspital, Bern University Hospital, University of Bern, Bern, Switzerland; u Department of Radiation Oncology, Clinique de La Source, Lausanne, Switzerland; v Department of Radiation Oncology, Hirslanden Clinique des Grangettes, Chêne-Bougeries, Switzerland; w Department of Radiation Oncology, Centre Hospitalier Universitaire Vaudois, Lausanne, Switzerland; x Department of Radiation Oncology, Réseau Hospitalier Neuchâtelois, La Chaux-de-Fonds, Switzerland

**Keywords:** Stereotactic radiotherapy, Brain metastases, Treatment planning, Dosimetric intercomparison, Quality assurance

## Abstract

**Background and purpose:**

Stereotactic radiotherapy (SRT) is a standard approach for treating multiple brain metastases. However, variation in planning practices may impact treatment quality. This study assessed planning consistency and dose–volume–based outcomes across radiation oncology centers.

**Materials and methods:**

A Computed Tomography (CT) scan of an anthropomorphic phantom with structure set was distributed to participating centers. Each center created SRT plans as for a clinical case. Dose distributions were evaluated based on Planning Target Volume (PTV) coverage (V_100_% (PTV)), dose to 95% of Gross Target Volume (GTV) volume (D_95_% (GTV)), maximal PTV dose (D_max_), conformity index (CI), gradient index (GI), brain volume receiving different percentages of the prescribed dose, and doses delivered to 0.035 cm^3^ and 0.5 cm^3^ of the brainstem.

**Results:**

Twenty-four centers, using 30 treatment units, submitted plans. The V_100_% (PTV) ranged from 95% to 100%, with D_max_ between 110% and 150% of the prescribed dose. Mean GTV dose ranged from 110% to 135%, and 81% of GTVs had D_95_% between 110% and 120%. High conformity was achieved in 74% of plans (CI < 1.1), while 67% had a GI between 3.4 and 5. All plans met clinical dose constraints for the brainstem and uninvolved brain.

**Conclusion:**

This interinstitutional comparison demonstrated high plan quality and adherence to critical organ constraints, despite variability in planning strategies. These findings support nationwide planning and quality assurance standards to ensure consistently high-quality SRT.

## Highlights

• Target coverage in brain stereotactic planning is more than 95% across 24 centers.
• Seventy four percent of plans showing high conformity with conformity index < 1.1.
• All plans met brainstem dose limits, and non-coplanar plans reduced low-dose brain.

## Graphical abstract

**Figure:**
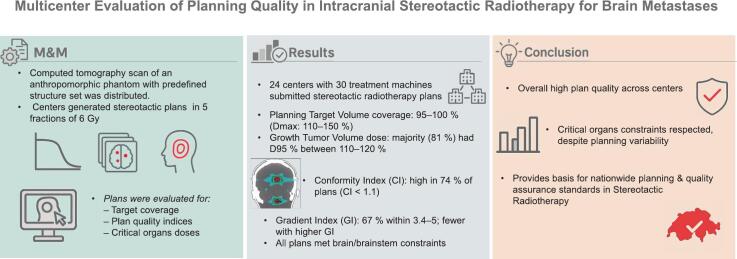

## 1 Introduction

Brain metastases are the most common adult intracranial malignancy, affecting a large proportion of patients with advanced cancer and representing a major cause of neurological morbidity and mortality [1], [2]. Stereotactic radiotherapy (SRT) delivers high-dose, conformal radiation in a single or few fractions with steep dose fall-off, making it particularly well-suited for these lesions [[3], [4]]. The overarching planning goal is to achieve precise target coverage while minimizing exposure to surrounding healthy tissues, as differences in these parameters can directly influence local control and toxicity rates.

In intracranial radiosurgery, steep dose gradients at the planning target volume (PTV) boundary are both a technical hallmark and a clinical necessity, especially when targets are close to critical structures. Plan quality is often characterized by conformity, how closely the high-dose region matches the target, quantified by the Conformity Index (CI) [5], and dose fall-off, described by the Gradient Index (GI) [6], [7]. Optimizing these metrics helps reduce unnecessary normal tissue exposure and supports safer, more effective treatment.

Delivering such precision requires rigorous quality assurance (QA), not only at the individual plan verification stage [8], [9], [10] but also through broader initiatives such as dosimetry audits and interinstitutional comparisons [11]. These programs benchmark plan quality, identify variations in technology use, dose prescription, and optimization strategies, and provide a foundation for clinical collaboration and trial participation [12]. Despite the widespread adoption of SRT, substantial variability in planning approaches persists across institutions, highlighting the need to better understand, and where possible harmonize, practice to ensure equitable, high-quality care nationwide [13], [14].

Building upon a previous national intercomparison effort focused on delivery accuracy, this study aimed to benchmark current SRT planning practices across centers. Interinstitutional variability in plan quality, dose conformity, and normal-brain sparing was quantified across different treatment platforms, collimation systems, and beam geometries to identify areas that may benefit from harmonization.

## 2 Materials and methods

### 2.1 Study design and phantom description

This analysis was undertaken as part of a national dosimetric intercomparison study using the anthropomorphic 3D-printed RTsafe Prime head phantom (RTsafe, Athens, Greece), coordinated by the University Hospital Zurich.

Detailed information on the phantom setup, imaging protocol, predefined target geometry, and delineated critical organs, as well as the full list of participating centers, treatment platforms, treatment planning systems (TPS) with dose-calculation algorithms, is provided in the Supplementary Material (Section A and B). Briefly, all centers received an identical CT dataset of a homogeneous head phantom with three predefined target volumes and selected critical organs, ensuring consistent geometry for interinstitutional comparison. Each center created SRT plans prescribing 6.0 Gy to all targets according to its local clinical protocol.

The Digital Imaging and Communications in Medicine (DICOM) dose files provided by each department were imported into a plan in the Eclipse treatment planning system (Eclipse 16.01.10, Varian Medical Systems, Inc., Palo Alto, CA), for evaluation purposes. All centers normalized the plans to > 96% of the PTV volume covered by the prescribed dose except two centers who normalized to the mean PTV dose. These two centers were evaluated separately.

### 2.2 Treatment planning and dosimetric evaluation

Thirty stereotactic radiotherapy (SRT) plans were submitted by 24 Swiss radiotherapy centers; three plans were excluded (see Supplementary Materials Section C). The study included different treatment platforms, comprising C-arm linear accelerators (linacs), CyberKnife systems (Accuray Incorporated, Sunnyvale, CA, USA), and Radixact systems (Accuray Incorporated, Sunnyvale, CA, USA), with various beam collimation devices, including multileaf collimators (MLCs), image-guided robotic radiosurgery (IRIS), and cones. The distribution of TPSs and beam collimation systems across the submitted plans is summarized in Table 1.

**Table 1:** Distribution of treatment planning systems and beam collimation systems across submitted plans.

Category	System	Number of plans	Percentage (%)
Treatment planning system	Eclipse	19	64
	RayStation	5	17
	Precision	4	13
	Monaco	1	3
	Pinnacle	1	3
Beam collimation system	Linac with 5 mm MLC	16	53.3
	Linac-2.5 mm MLC	7	23.3
	Robotic radiosurgery-IRIS	3	10
	Helical tomotherapy-6.25 mm MLC	2	6.7
	Robotic radiosurgery-Cone	2	6.7

The dose distribution was evaluated using several metrics: the volume of the PTV receiving the prescribed dose (V_100_% (PTV)), the dose delivered to 95% of the GTV (D_95_% (GTV)) volume, the maximum dose to the PTV (D_max_), the conformity index and the gradient index. The conformity index was calculated according to the Radiation Therapy Oncology Group (RTOG) definition (CI = V_RI_ / V_TV_), where V_RI_ is the volume receiving the prescription dose and V_TV_ is the target volume [15]. The gradient index was defined as GI = V_50%_ / V_100%,_ representing the ratio of the volume receiving 50% of the prescription dose to the volume receiving 100%. Additionally, the brain volumes receiving 30.0 Gy (100%), 25.0 Gy (83.3%), 20.0 Gy (66.6%), 15.0 Gy (50%), 10.0 Gy (33.3%), and 5.0 Gy (16.6%) were assessed across different treatment platforms, coplanar and non-coplanar beam arrangements, and single-isocenter versus multi-isocenter techniques.

Specific dose-volume metrics were also extracted to assess the dose delivered to critical organs, with a focus on the brainstem and normal brain tissue. For each submitted plan, the maximum dose to 0.035 cm^3^ (D_0.035 cm_^3^_)_ and the dose to 0.5  cm^3^ (D_0.5 cm_^3^) of the brainstem were recorded, in accordance with the American Association of Physicists in Medicine (AAPM) TG-101 recommendations for five-fraction stereotactic radiotherapy [16].

To assess the exposure of uninvolved brain tissue to intermediate and high doses, three additional dose-volume metrics were evaluated, which are increasingly recognized as predictors of toxicity. These were the volumes of normal brain receiving at least 25.0 Gy (BrainV_25 Gy_), 30.0  Gy (BrainV_30 Gy_), and 28.8  Gy (BrainV_28.8 Gy_). Based on thresholds proposed in the literature, BrainV_25_ should remain below 16.0  cm^3^, BrainV_30 Gy_ between 10.5 and 30.0  cm^3^, and BrainV_28.8 Gy_ below 7.0  cm^3^ to minimize the risk of adverse events in healthy brain tissue [17], [18]. These metrics were extracted for all submitted plans using a five-fraction regimen across participating institutions.

To complement this analysis and evaluate the low-dose spread within healthy brain regions, the volume of brain minus GTV receiving ≥ 4.8 Gy per fraction (V_4.8 Gy_) was also calculated. This threshold was selected as the radiobiological equivalent of the commonly cited V_12 Gy_ constraint for single-fraction treatments, assuming an α/β ratio of 2.0  Gy.

### 2.3 Statistical analysis

Statistical analyses were performed using Python (version 3.10) with the SciPy library (version 1.12.0). For pairwise comparisons between two independent groups, such as coplanar versus non-coplanar or single-isocenter versus multi-isocenter techniques, the non-parametric Mann–Whitney *U* test was applied. For comparisons involving more than two groups, the Kruskal–Wallis test was used, followed by Bonferroni-adjusted post-hoc analysis when statistically indicated. Exact p-values are reported, and a p-value < 0.05 was considered statistically significant.

## 3 Results

**Fig. 1:**
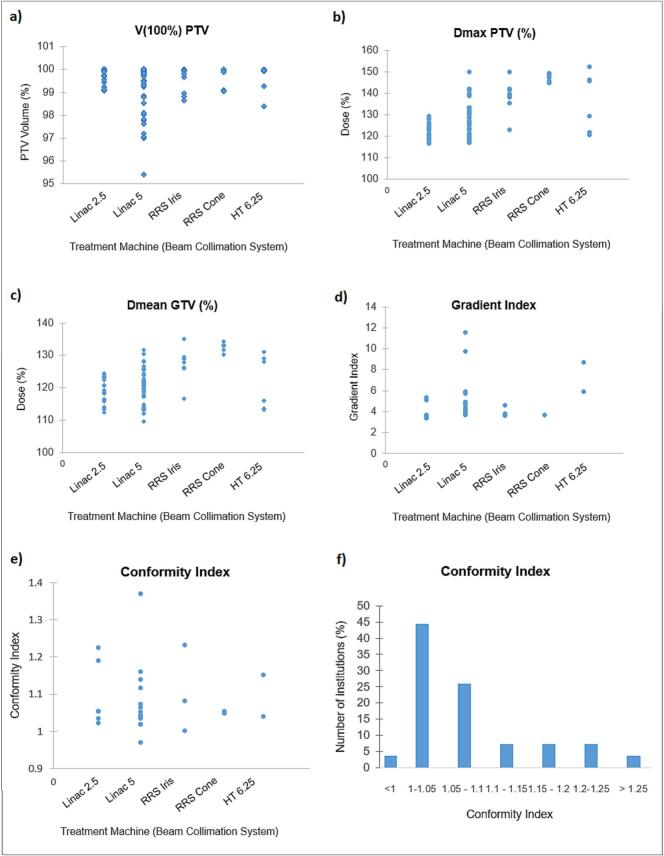
Distribution of key plan quality and dose-volume metrics across institutions and treatment platforms, including PTV coverage, maximum and mean target doses, and conformity and gradient indices by machine and collimation system. Treatment machine and beam collimation systems: linac with 2.5-mm MLC, linac with 5-mm MLC, robotic radiosurgery with IRIS collimation (RRS- IRIS), robotic radiosurgery with fixed cone collimation (RRS-Cone), and helical tomotherapy with 6.25-mm MLC (HT-6.25).

Except for one outlier, all treatment plans achieved coverage of ≥97% of the PTV by the prescribed dose, regardless of collimation type. As shown in Fig. 1a, more than half of the plans reached V_100_% ≥ 99%, with the remainder predominantly 97–99%. Differences in normalization across centers produced variation in maximum PTV dose; Fig. 1b shows D_max_ spanning ∼110–150% of prescription, with most plans between 117 and 133%. This pattern was consistent with Fig. 1c, which indicates mean GTV doses clustered around 120–130% (overall 110–135%), reflecting the intentional intratumoral hotspots commonly used in SRT to support tumor control.

Plan conformity was generally high, with 74% of cases having CI < 1.1 and 89% below 1.2. Gradient index values showed wider variation: approximately two-thirds were between 3.4 and 5.0, while one-third exceeded 5.0, indicating less steep dose fall-off. Median GI was lowest for linacs with 2.5 mm MLCs and highest for helical tomotherapy, with non-coplanar beam arrangements tending to achieve higher conformity and steeper gradients.

Platform-specific gradient behavior is shown in Fig. 1d: GI medians ranged from 3.6 for linacs with 2.5 mm MLCs to 7.3 for helical tomotherapy. Robotic radiosurgery, using the IRIS platform, exhibited the tightest interquartile range (0.2), while linac with 5 mm MLC showed the widest (2.6). Fig. 1e shows the CI distribution by platform, with medians tightly clustered (∼1.1–1.3), indicating minimal inter-platform variation in target conformity. The overall institutional CI distribution is summarized in Fig. 1f, with 74% of plans having CI < 1.1 and 89% < 1.2. Dose fall-off varied more at the institutional level (Fig. 1g): approximately two-thirds of plans achieved GI 3.4 – 5.0, while about one-third exceeded 5.0, indicating less steep gradients. Non-coplanar beam arrangements tended to achieve higher conformity and steeper gradients.

All plans met clinical dose constraints for the brainstem and uninvolved brain. Mean brainstem D_0.035_ cm^3^ was 18.2 Gy (range 11.5–26.0 Gy) and D_0.5_ cm^3^ averaged 9.9 Gy (range 5.0–17.5 Gy). BrainV_25 Gy_, BrainV_28.8 Gy_, and BrainV_30 Gy_ remained well below published toxicity thresholds, with mean values of 10.6 cm^3^, 4.5 cm^3^, and 3.6 cm^3^, respectively. The mean V_4.8 Gy_ for the whole brain (including targets) was 6.9 cm^3^ (range 5.5–10.9 cm^3^).

**Fig. 2:**
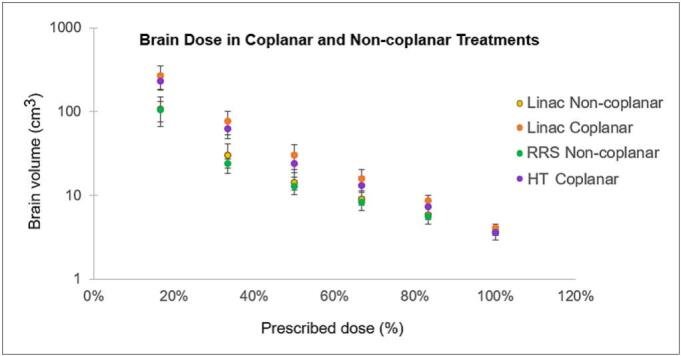
Brain volume (cm^3^) receiving 17%–100% of the prescription dose by platform and geometry. Linac non-coplanar (yellow), linac coplanar (orange), Robotic radiosurgery (non-coplanar) (green), helical tomotherapy coplanar (purple). Lower low-/intermediate-dose spread is evident with non-coplanar delivery. The y-axis is displayed on a logarithmic scale. (For interpretation of the references to colour in this figure legend, the reader is referred to the web version of this article.)

**Fig. 3:**
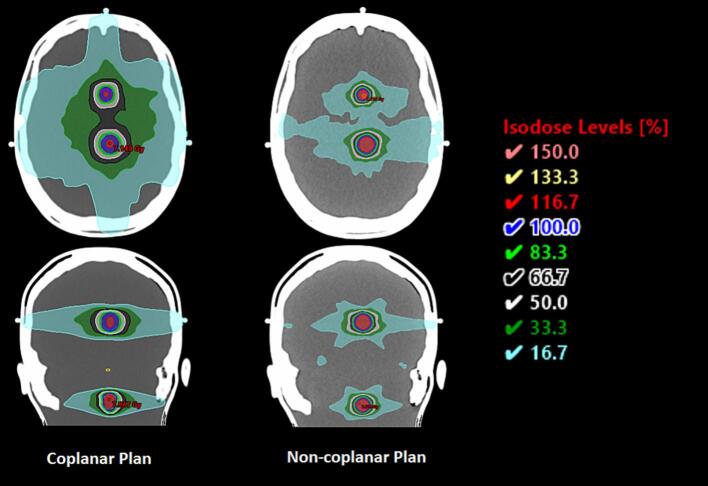
Representative coplanar vs non-coplanar dose distributions on two linacs with 5 mm MLCs, illustrating reduced peripheral dose with non-coplanar geometry.

Non-coplanar plans demonstrated reduced low to intermediate dose spread compared with coplanar plans (Fig. 2). This effect is visually exemplified in Fig. 3, which shows coplanar and non-coplanar dose distributions for linac plans with 5 mm MLCs, illustrating the reduced peripheral dose achieved with non-coplanar geometry.

**Fig. 4:**
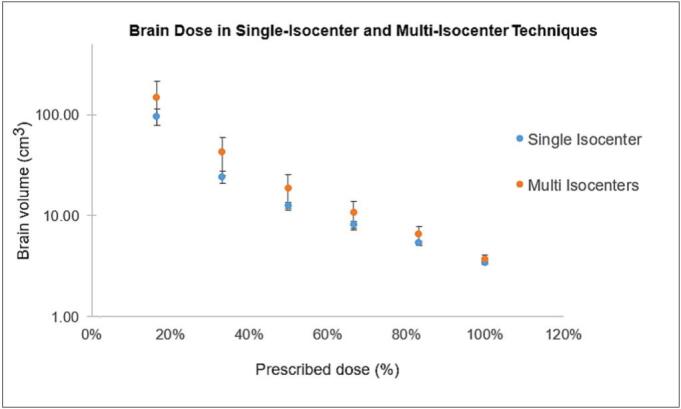
Brain volume (cm^3^) receiving 17%–100% of the prescription dose by isocenter technique. Single-isocenter multi-target (blue) and multi-isocenter (orange) plans are compared across all participating institutions (excluding robotic radiosurgery and helical tomotherapy). The y-axis is displayed on a logarithmic scale. (For interpretation of the references to colour in this figure legend, the reader is referred to the web version of this article.)

As illustrated in Fig. 4, single-isocenter plans yielded consistently lower brain volumes at different dose levels compared to multi-isocenter plans.

**Fig. 5:**
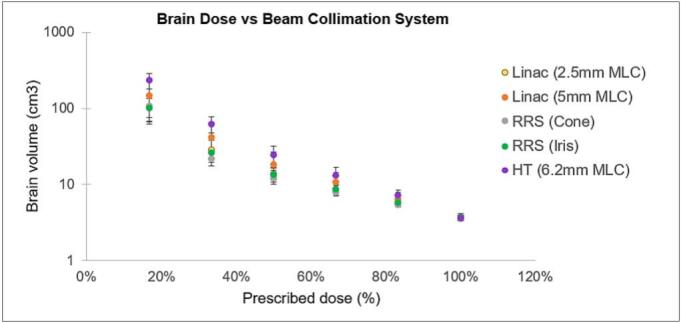
Brain volumes (cm^3^) receiving 17%–100% of the prescription dose by collimation system: linac 2.5 mm MLC (yellow), linac 5 mm MLC (orange), robotic radiosurgery fixed cone (gray), robotic radiosurgery IRIS (green), helical tomotherapy 6.2 mm MLC (purple). The y-axis is displayed on a logarithmic scale. (For interpretation of the references to colour in this figure legend, the reader is referred to the web version of this article.)

Fig. 5 provides a graphical overview of brain dose–volume relationships across collimation systems (color legend as indicated). The corresponding numerical data, including detailed comparisons among different treatment platforms, coplanar versus non-coplanar linac deliveries, and single- versus multi-isocenter linac techniques, are presented in Supplementary Table S.1.

For coplanar (n = 5) versus non-coplanar (n = 16) plans, no significant difference was observed for target coverage at V_30 Gy_ (median 3.8 vs 3.5 cm^3^; p = 0.18). In contrast, non-coplanar plans showed significantly lower low- and intermediate-dose brain exposure, with reductions observed from V_25 Gy_ through V_5 Gy_ (all p ≤ 0.02).

Similarly, single-isocenter (n = 6) and multi-isocenter (n = 14) plans did not differ significantly for V_30 Gy_ (p = 0.16), while single-isocenter plans exhibited lower brain volumes receiving intermediate and low doses. After Bonferroni correction, differences remained statistically significant for V_15 Gy_ (p = 0.03), V_10 Gy_ (p = 0.02), and V_5 Gy_ (p = 0.01), whereas V_25 Gy_ was no longer significant (p = 0.10) and V_20 Gy_ remained borderline (p = 0.05).

Across beam collimation systems, no significant differences were detected for brain dose–volume metrics (V_30 Gy_ p = 0.78; V _5 Gy_ –V_15 Gy_ p = 0.08–0.12); a non-significant trend toward lower V_10 Gy_ –V_15 Gy_ was seen with Robotic radiosurgery and linac 2.5 mm MLC, and higher values with helical tomotherapy. For conformity metrics, GI differed by platform (p = 0.03), although no pairwise comparisons remained significant after correction. Conformity index values were comparable across systems, with median values ranging from 1.1 to 1.3.

## 4 Discussion

We evaluated 27 stereotactic radiotherapy plans from 24 Swiss centers, representing ∼90% of national departments. Intracranial SRT planning was consistently high quality: nearly all plans achieved ≥ 97% PTV coverage with intentional intratumoral hotspots (mean GTV doses ∼120–130% of prescription). Most plans achieved excellent conformity (CI < 1.1 in 74%), whereas GI showed wider dispersion, with one-third of plans exceeding a value of 5, indicating greater variability in dose fall-off and normal-brain exposure.

Non-coplanar arrangements were associated with differences in plan quality metrics, while all brainstem and normal-brain dose constraints remained within safety thresholds. These findings supported standardizing plan reporting (CI, GI, D_max_/PTV, mean GTV, Vx equivalents) and aligning normalization practices to reduce inter-center variability.

The International Stereotactic Radiosurgery Society reports wide variability in prescription isodose lines (20–90%, median 50%) [19], emphasizing the absence of a universal standard. Lower prescription isodoses (50–80%) improve tumor control, conformity, and toxicity [20], [21], [22], [23], [24], [25], [26]. Our findings suggest that, while Swiss centers reliably achieve high coverage, systematic reporting of the chosen prescription isodose line, alongside D_max_/PTV and mean GTV, would aid practice harmonization without constraining patient-specific adaptation.

Documenting both high-dose shaping and low-/intermediate-dose spread remains essential: target conformity links to local control, while low-dose spill correlates with radionecrosis risk [6], [27], [28]. Differences across centers likely stem from normalization practices; refining these may improve overall plan quality. When non-coplanar arrangements were used, improved conformity and steeper dose gradients were observed, resulting in reduced normal-brain exposure. Given that GI values greater than 5.0 have been associated with increased toxicity, this highlights the clinical relevance of achieving low GI where feasible [27], [29], [30].

For conformity-related measures, the overall Kruskal–Wallis test indicated a statistically significant difference in GI among platforms (p = 0.032); however, no pairwise differences remained significant after Bonferroni correction. As shown in Fig. 1d–1g, GI medians varied modestly, with most values tightly grouped across institutions and a few higher values observed for helical tomotherapy and certain linac 5-mm MLC cases. In contrast, CI was broadly comparable between systems (Kruskal–Wallis p = 0.10), with medians clustered around 1.1–1.3 and 74% of plans < 1.1 and 89% < 1.2, confirming consistently high conformity. Overall, these results indicated that, despite platform-specific geometric and collimation differences, centers applied similar optimization priorities and achieved uniform target conformity, while remaining variations in gradient behavior likely reflect differences in normalization approach and achievable non-coplanarity.

All submitted plans met brainstem constraints, a critical consideration given its radiosensitivity. AAPM TG-101 recommends D_0.035 cm3_ ≤ 31.0 Gy and D_0.5 cm3_ < 23.0 Gy for five fractions [16], consistent with QUANTEC guidance and reiterated in ASTRO’s 2022 SRS/SBRT safety white paper and model policies [31], [32]. For the normal brain, published guidance recommends limiting maximum point dose and dose-volume metrics such as V_12 Gy_ in single-fraction SRS to reduce radionecrosis risk [31], [32], [33], [34], [35]. In our five-fraction setting, the biologically equivalent.

V_4.8 Gy_ (assuming α/β = 2.0 Gy) remained within safe thresholds for all plans. The consistently low BrainV_25 Gy_, BrainV_28.8 Gy_, and BrainV_30 Gy_ values, frequently below published limits, further highlight effective healthy-tissue sparing among participating centers [34], [36].

Cross-platform comparisons revealed that smaller MLC leaf widths (2.5 mm) and robotic radiosurgery cone/IRIS collimation achieved steeper dose gradients and higher conformity. Non-coplanar delivery reduced low- to intermediate-dose brain volumes (V_5 Gy_ –V_25 Gy_) without affecting target coverage (V_30 Gy_), supporting its geometric advantage in multi-target SRT [29]. Across collimation systems, differences in low-dose spread were not statistically significant, though a trend toward lower V_10 Gy_ –V_15 Gy_ was observed for robotic radiosurgery and 2.5 mm MLC plans, and higher values for helical tomotherapy. Among C-arm linac plans, single-isocenter approaches showed significantly lower V_20 Gy_ –V_5 Gy_ than multi-isocenter techniques, while maintaining comparable V_25 Gy_ and V_30 Gy_, reflecting the benefit of unified optimization and tighter overall dose control. These findings, consistent across low-dose levels even after Bonferroni adjustment, indicated a dosimetric advantage of single-isocenter planning for dose compactness in multi-target SRT [37], [38].

For shaping metrics, GI differed overall by platform but lacked significant pairwise differences after correction, while CI was broadly comparable across systems (median ∼1.1–1.3). Given the heterogeneity in planning objectives and geometries, these findings should be interpreted as hypothesis-generating. Specifically, they suggest that the availability of finer collimation and the use of non-coplanar arrangements may contribute to improved plan quality, warranting further systematic investigation.

This study intentionally allowed each participating center to apply its own institutional stereotactic planning protocol, mirroring routine clinical practice. This design enabled the assessment of real-world interinstitutional variability and provided a representative picture of current Swiss SRT planning. Such an approach captures both the strengths and diversity of national practice, offering a foundation for future harmonization efforts.

These findings should be viewed in light of certain study limitations. The analysis was restricted to the brainstem and whole brain; other critical organs were not assessed due to their anatomical distance from the target volumes in the phantom model. Only a single patient case was planned, limiting the representation of clinical diversity. This audit included three target sizes representative of typical brain metastases, which were predominantly spherical. While this reflects common clinical presentations, future audits could explore additional scenarios such as irregularly shaped targets or lesions in anatomically challenging locations, alongside an expanded evaluation of other critical organs, to further test planning versatility. Furthermore, heterogeneity in treatment planning systems, dose calculation algorithms, and institutional preferences introduced variability that, while reflecting real-world practice, constrains the ability to establish universal planning benchmarks.

In addition, the present audit did not include direct reporting of V_12 Gy_ for single-fraction SRS, a widely used international benchmark, instead assessing its 5-fraction equivalent (V_4.8 Gy_). While this approach is appropriate for the fractionation used, it may limit direct comparison with single-fraction datasets. Future audits could address this by reporting V_12 Gy_ for single-fraction plans alongside the appropriate biologically equivalent values for multi-fraction regimens, thereby facilitating comparison with other studies and aligning national benchmarking with widely recognized international reporting standards.

While the current study does not assess clinical outcomes directly, the differences in calculated dose distributions, particularly in CI, GI, and dose heterogeneity, warrant cautious consideration. Given that all centers achieved adequate PTV coverage and respected critical organs limits, these variations are unlikely to compromise patient safety in most cases. However, in anatomically complex cases, subtle differences in plan normalization, conformity, or dose fall-off could influence long-term local control or toxicity. These results highlighted the value of interinstitutional comparisons not only for benchmarking but also for fostering ongoing dialogue on clinical impact.

Overall, the findings of this study meet its objective of benchmarking stereotactic radiotherapy planning practices across Swiss centers and quantifying interinstitutional variability. Variations were primarily observed in gradient index, dose normalization, and the extent of low-dose brain exposure across platforms and beam geometries. These insights highlighted concrete opportunities for harmonization, particularly through standardized plan normalization, systematic reporting of conformity and gradient indices, and the broader adoption of non-coplanar and single-isocenter strategies when feasible.

In conclusion, this national interinstitutional comparison shows that Swiss centers consistently achieve high-quality intracranial SRT plans with excellent target coverage and adherence to critical organs constraints. Variability in conformity, dose gradients, and normalization practices highlights opportunities for further harmonization. Standardized reporting of plan metrics and broader use of optimized beam arrangements, such as non-coplanar techniques, may further strengthen SRT planning consistency and support future multicenter clinical trials.

## Declaration of competing interest

The authors declare the following financial interests/personal relationships which may be considered as potential competing interests: The University Hospital Zurich holds research and teaching agreements with Varian/Siemens Healthineers, which may be considered a potential conflict of interest. However, these agreements had no influence on the design, execution, or interpretation of this study.
