# Supplementary material for: Multicenter evaluation of planning quality in intracranial stereotactic radiotherapy for brain metastases
Source: Phys Imaging Radiat Oncol. 2026 Feb 6;37:100919. doi: 10.1016/j.phro.2026.100919 (PMC12914438; doi:10.1016/j.phro.2026.100919)
Supplement: Supplementary Data 1 [file mmc1.pdf]

## Supplementary Materials

### A. Phantom Description, Imaging, and Target Definition

The RTsafe Prime head phantom (RTsafe P.C., Athens, Greece) is water-filled and designed to accommodate various radiation detectors for accurate dose measurement and plan verification. A 3D CT scan of the phantom, along with a delineated structure set, was provided to each radiotherapy center. The scan was acquired on a Siemens Somatom Edge Plus CT scanner (Siemens AG, Erlangen, Germany) with a standard clinical imaging protocol (120 kV, 430 mAs, slice thickness 0.6 mm, medium-smooth Br38 kernel for reconstruction).

The PTVs (0.5 $cm^3$, 1 $cm^3$, and 2 $cm^3$) were predefined in the reference CT dataset as 1 mm symmetric isotropic expansions of the corresponding GTVs (brain metastases (BMs)). These structures were distributed to all participating centers to ensure uniform target geometry across institutions. The first two targets were positioned on the same coronal plane, while the third target was aligned with the axial plane of the first (Figure S.1). Additionally, various critical organs were delineated, including the brainstem, optic chiasm, bilateral eyes, optic nerves, and lenses.

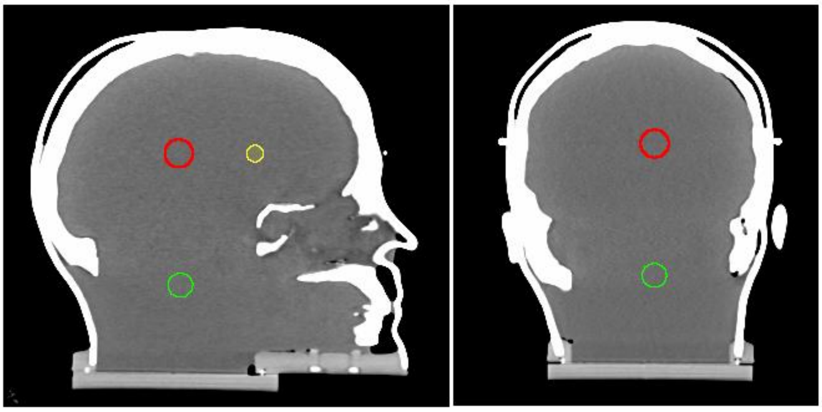

Figure S.1. Brain targets on the homogeneous phantom. BM1 (red) has a diameter of 1.5 cm, BM2 (yellow) has a diameter of 0.5 cm, and BM3 (green) has a diameter of 1cm.

### B. Planning Instructions and Participating Centers

Each center was requested to develop a treatment plan prescribing 6 Gy to all designated targets, following their respective clinical protocols. This dose was selected primarily to ensure accurate film dosimetry with EBT3 films and to keep the recorded dose on the EBT3 films below 10 Gy. Additionally, this fractionation is commonly used in SRT for brain metastases [1-3].

Thirty plans were initially submitted from 24 Swiss radiotherapy centers. Devices and treatment planning systems(TPS)/dose-calculation algorithms included: 20 Varian linacs planned in Eclipse with Acuros; one Elekta linac planned in Monaco (Monte Carlo); one Elekta linac planned in RayStation (Monte Carlo); one Elekta Synergy planned in Pinnacle (Collapsed Cone Convolution, CCC); three CyberKnife systems planned in Precision (ray tracing); two CyberKnife systems planned in RayStation (CCC); one Radixact planned in Precision (Fluence-Convolution Broad-Beam, FCBB); and one helical tomotherapy planned in RayStation (CCC). All TPS used type-B dose-calculation algorithms. Collimation comprised linac MLCs with 2.5

mm (n=7) or 5 mm (n=16) leaf widths, Radixact MLC 6.25 mm (n=2), and CyberKnife cone (n=2) or IRIS (n=3). Beam arrangements included: eight C-arm linac centers using a two-isocenter coplanar arc technique; eight using two or three isocenters with multiple arcs and couch angles; and seven using a single-isocenter approach with multiple arcs and couch angles. Radixact plans used two-isocenter coplanar delivery; CyberKnife employed non-coplanar robotic beam arrangements.

### C. Plan Review and Quality Screening

After quality screening (i.e., visual inspection of imported dose distributions to confirm dataset completeness and consistency of dose scaling), three submissions were excluded: in one plan, one of the predefined targets had been omitted, and in two plans, the optimization aimed for homogeneous PTV dose distributions rather than stereotactic dose heterogeneity. The final dosimetric analysis therefore included 27 plans.

| Brain Dose-Volume Data | | | | | | |
|---|---|---|---|---|---|---|
| | $V_{30Gy}$ | $V_{25Gy}$ | $V_{20Gy}$ | $V_{15Gy}$ | $V_{10Gy}$ | $V_{5Gy}$ |
| Linac (2.5mm MLC) | 3.7 ± 0.3 [3.6 (3.5–3.9)] | 5.9 ± 0.8 [5.5 (5.3–6.3)] | 8.7 ± 1.5 [8.1 (7.7–9.8)] | 13.9 ± 2.8 [12.7 (11.8–16.17)] | 28.7 ± 9.13 [24.5 (22.8–34.9)] | 105.3 ± 28.9 [116 (80–128.5)] |
| Linac (5mm MLC) | 3.7 ± 0.3 [3.6 (3.5–3.8)] | 6.5 ± 1.4 [5.9 (5.7–6.9)] | 10.7 ± 3.6 [9.5 (8.2–10.7)] | 18.4 ± 8.4 [15.2 (13.4–19.4)] | 41.4 ± 23.9 [32.1 (25.2–49.6)] | 146.5 ± 84.2 [114.5 (95.3–175.4)] |
| CyberKnife (Iris) | 3.7 ± 0.4 [3.7 (3.5–4.0)] | 5.8 ± 0.7 [5.6 (5.4–6.2)] | 8.7 ± 1.3 [8.3 (8.1–9.2)] | 13.5 ± 1.8 [12.8 (12.6–14.2)] | 25.9 ± 3.3 [25.9 (24.2–27.6)] | 101.53 ± 33.7 [112.8 (88.2–120.5)] |
| CyberKnife (Cone) | 3.6 ± 0.0 [3.6 (3.6–3.6)] | 5.4 ± 0.1 [5.4 (5.3–5.5)] | 7.9 ± 0.2 [7.9 (7.9–8.0)] | 12.0 ± 0.5 [12.1 (11.9–12.2)] | 21.7 ± 0.6 [21.7 (21.5–21.9)] | 109.1 ± 42.0 [109.1 (94.2–124.0)] |
| Radixact (6.2mm MLC) | 3.6 ± 0.3 [3.6 (3.5–3.7)] | 7.3 ± 1.1 [7.3 (6.9–7.7)] | 13.2 ± 3.6 [13.1 (11.9–14.4)] | 24.3 ± 7.6 [22.4 (18.7–26.0)] | 62.6 ± 15.0 [61.9 (56.2–67.5)] | 234.0 ± 53.7 [233.9 (215.0–252.9)] |
| Non-coplanar (Linacs) | 3.6 ± 0.2 [3.5 (3.5–3.6)] | 5.9 ± 0.6 [5.7 (5.5–5.9)] | 8.9 ± 1.1 [8.7 (8.1–9.9)] | 14.3 ± 2.5 [13.4 (12.6–16)] | 29.6 ± 9.3 [25.2 (22.9–32.3)] | 106.2 ± 32.1 [98.1 (80.3–117.4)] |
| Coplanar (Linacs) | 4.1 ± 0.5 [3.9 (3.8–4.1)] | 8.1 ± 1.5 [7.8 (7.4–8.6)] | 14.5 ± 4.5 [13.2 (11.4–16.3)] | 27.9 ± 9.8 [26.5 (19.8–34.5)] | 71.3 ± 22.1 [70.7 (54–88.2)] | 260.4 ± 70.7 [266.7 (217.5–309.6)] |
| Single Isocenter (Linac) | 3.5 ± 0.1 [3.5 (3.3–3.6)] | 5.4 ± 0.2 [5.4 (5.1–5.8)] | 8.2 ± 0.8 [8.1 (7.4–9.1)] | 12.7 ± 1.2 [12.6 (11.4–15.2)] | 24.8 ± 3.5 [22.9 (21.9–32.3)] | 98.4 ± 17.5 [99.8 (78.5–117.4)] |
| Multi Isocenters (Linac) | 3.8 ± 0.3 [3.7 (3.5–4.6)] | 6.7 ± 1.3 [6.0 (5.5–10.3)] | 10.9 ± 3.3 [9.8 (8.0–20.9)] | 19.0 ± 6.9 [16.2 (12.3–39.2)] | 43.2 ± 17.5 [38.6 (22.5–94.0)] | 149.6 ± 69.7 [132.2 (70.9–332.6)] |

Table S.1. Brain dose–volume metrics across treatment platforms and beam geometries. The table summarizes V5–V30 Gy values for different collimation systems, including subgroup comparisons for coplanar versus non-coplanar linac deliveries and single- versus multi-isocenter linac techniques.

## References


[1]. Gruber I, Stark P, Weidner K, Treutwein M, Koelbl O. Fractionated stereotactic radiotherapy of brain metastases: results of a retrospective study. Radiat Oncol. 2023; 18:85. https://doi.org/10.1186/s13014-023-02277-6

[2]. Piras A, Boldrini L, Menna S, Sanfratello A, D'Aviero A, Cusumano D, et al. Five-fraction stereotactic radiotherapy for brain metastases: a single-institution experience on different dose schedules. Oncol Res Treat. 2022; 45:408–14. https://doi.org/10.1159/000522645

[3]. Rogers S, Stauffer A, Lomax N, Alonso S, Eberle B, Gomez Ordonez S, et al. Five fraction stereotactic radiotherapy after brain metastasectomy: a single-institution experience and literature review. J Neurooncol. 2021; 155:35–43. https://doi.org/10.1007/s11060-021-03840-5
